# Supplementary material for: Highly Efficient Cardiac Differentiation and Maintenance by Thrombin-Coagulated Fibrin Hydrogels Enriched with Decellularized Porcine Heart Extracellular Matrix
Source: Int J Mol Sci. 2023 Feb 2;24(3):2842. doi: 10.3390/ijms24032842 (PMC9917900; doi:10.3390/ijms24032842)
Supplement: Supplementary file 1 [file ijms-24-02842-s001.zip › S9.pdf]

Supplementary S9: Co-culture of H9c2 and Nor-10 Fibroblasts in the presence and absence of retinoids

A

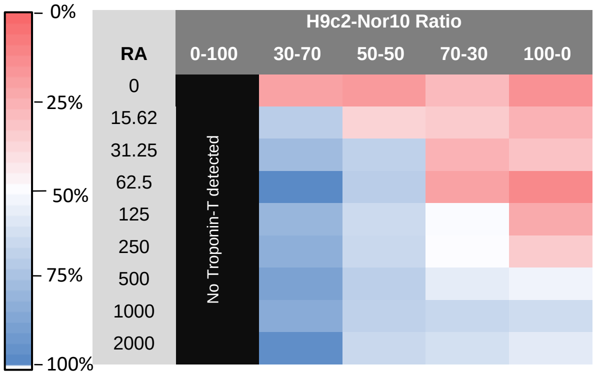

B

Retinoic acid Concentration(nM)	*H9c2/ Fibroblast cells*	
	**30/70**	**100/0**
0	7±0.87	5±0.69
62.5	81±11.89	4±1.03
2000	79±10.43	28±5.71

Figure S9-1. Differentiation of H9c2 cells in mono- and co-culture on tissue culture plates. A) The heat map of normalized differentiation percentage of H9c2 cells for different concentrations of retinoic acid and for different ratios of H9c2 to fibroblast cells. Cultures in 2D, without hydrogel, for 7 days (first 5days low serum). Retinoic acid concentration in nM. B) Selected numerical values for the differentiation percentages for the 30/70 ratio and 100/0 ratio.

Figure S9-1 summarizes graphically the differentiation percentage of H9c2 cells (evaluated as in the main text). These preliminary experiments were carried out on regular tissue culture plates to better quantify the effect of co-culture on H9c2 differentiation, and also to evaluate whether Three important conclusions can be drawn: First, in pure Nor-10 fibroblast cultures (“0-100” in Fig. S9-1A), no troponin T could be detected, regardless of the retinoic acid concentration. This indicates that cardiogenic differentiation is specific to the H9c2 cells. Second, in the absence of retoinoic

acid, cardiogenic differentiation remains always low on tissue culture plates, regardless of the co-culture conditions. Finally, co-culture with Nor-10 fibroblasts lowers the concentration of retinoic acid necessary to achieve efficient differentiation (in blue in Fig. S9-1A).

Overall, we see that on tissue culture plates, Nor-10 fibroblasts can partially substitute for addition of retinoic acid to induce cardiogenic differentiation in H9c2 cells. A minimal amount of retinoic acid (below 15nM) remains however necessary. This contrasts with the co-cultures on and in 3D hydrogels described in the main text, where efficient cardiogenic differentiation was obtained in the complete absence of exogeneous retinoic acid. This indicates that the combination of co-culture with Nor-10 fibroblasts and culture in the presence of various hydrogels completely substitutes for addition of retinoid acid.
