# Supplementary material for: Highly Efficient Cardiac Differentiation and Maintenance by Thrombin-Coagulated Fibrin Hydrogels Enriched with Decellularized Porcine Heart Extracellular Matrix
Source: Int J Mol Sci. 2023 Feb 2;24(3):2842. doi: 10.3390/ijms24032842 (PMC9917900; doi:10.3390/ijms24032842)
Supplement: Supplementary file 1 [file ijms-24-02842-s001.zip › Supplement materials.pdf]

Here you can find the links to each supplementary file:

S1: https://drive.google.com/file/d/1BIgdEg2hB104PYTiSFIKCwpmUw2wK-1h/view?usp=sharing
S2:https://drive.google.com/file/d/1HeuR_2lAF0apVB_wkWRyf_SJVYbem5Vs/view?usp=sharing
S3: https://drive.google.com/file/d/1Q_aK8a5qam0bkRFlkGNZQq79Xu5FNtUh/view?usp=sharing
S4: https://drive.google.com/file/d/1770eMxpYRrdeuxW7eot4y7Lq0uEbW4US/view?usp=sharing
S5: https://drive.google.com/file/d/1QzKHVx3kg8z5llFzJNsUkPZQ836eKoe9/view?usp=sharing
S6: https://drive.google.com/file/d/19U3_5kI3JaJzjwXa9y3j6YTaC2EjSotu/view?usp=sharing
S7: https://drive.google.com/file/d/1TE4NydweAR_AtOviCXojTv_G1SBRHxII/view?usp=sharing
S8: https://drive.google.com/file/d/1o5oSj90jnKTL5QTeRpYG130WtlkKcBQL/view?usp=sharing
S9: https://docs.google.com/document/d/1nbfBWiogL5B9K1d6R4HWh5vBJWMgW1yQ/edit?usp=sharing&ouid=102686338225364134241&rtpof=true&sd=true
