# Supplementary figures and images for: Highly Efficient Cardiac Differentiation and Maintenance by Thrombin-Coagulated Fibrin Hydrogels Enriched with Decellularized Porcine Heart Extracellular Matrix
Source: Int J Mol Sci. 2023 Feb 2;24(3):2842. doi: 10.3390/ijms24032842 (PMC9917900; doi:10.3390/ijms24032842)

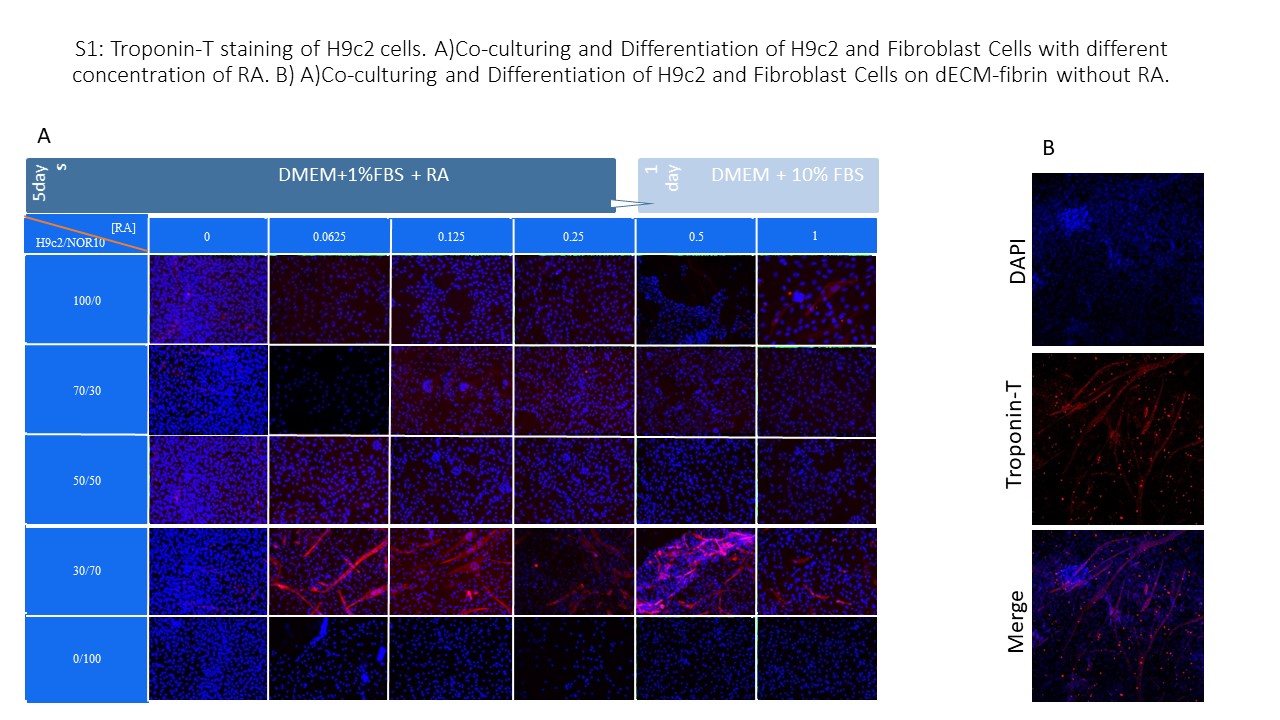

Supplement: Supplementary file 1 [file ijms-24-02842-s001.zip › S1 staining for cardiac Troponin T.jpg]
